# Exposure to Anacardiaceae Volatile Oils and Their Constituents Induces Lipid Peroxidation within Food-Borne Bacteria Cells

**DOI:** 10.3390/molecules17089728

**Published:** 2012-08-14

**Authors:** Ricardo M. Montanari, Luiz C. A. Barbosa, Antonio J. Demuner, Cleber J. Silva, Nelio J. Andrade, Fyaz M. D. Ismail, Maria C. A. Barbosa

**Affiliations:** 1 Department of Chemistry, Federal University of de Viçosa, Viçosa 36570-000, MG, Brazil; 2 Federal University of São João del’Rei, Campus Sete Lagoas, Sete Lagoas 35701-970, MG, Brazil; 3 Department of Food Technology, Federal University of de Viçosa, Viçosa 36570-000, MG, Brazil; 4 School of Pharmacy & Biomolecular Sciences, Institute for Health Research, Liverpool John Moores University, Byrom Street, Liverpool L3 3AF, UK; 5 Department of Nutrition, Federal University of Juiz de Fora, Juiz de Fora 36036-900, MG, Brazil

**Keywords:** essential oils, *δ*-3-carene, Anacardiaceae, antibacterial activity, lipid peroxidation

## Abstract

The chemical composition of the volatile oils from five Anacardiaceae species and their activities against Gram positive and negative bacteria were assessed. The peroxidative damage within bacterial cell membranes was determined through the breakdown product malondialdehyde (MDA). The major constituents in *Anacardium* *humile* leaves oil were (*E*)-caryophyllene (31.0%) and *α*-pinene (22.0%), and in *Anacardium occidentale* oil they were (*E*)-caryophyllene (15.4%) and germacrene-D (11.5%). Volatile oil from *Astronium fraxinifolium* leaves were dominated by (*E*)-*β*-ocimene (44.1%) and *α*-terpinolene (15.2%), whilst the oil from *Myracrodruon urundeuva* contained an abundance of *δ*-3-carene (78.8%). However, *Schinus terebinthifolius* leaves oil collected in March and July presented different chemical compositions. The oils from all species, except the one from *A. occidentale*, exhibited varying levels of antibacterial activity against *Staphylococcus aureus*, *Bacillus cereus* and *Escherichia coli*. Oil extracted in July from *S. terebinthifolius* was more active against all bacterial strains than the corresponding oil extracted in March. The high antibacterial activity of the *M. urundeuva* oil could be ascribed to its high *δ*-3-carene content. The amounts of MDA generated within bacterial cells indicate that the volatile oils induce lipid peroxidation. The results suggest that one putative mechanism of antibacterial action of these volatile oils is pro-oxidant damage within bacterial cell membrane explaining in part their preservative properties.

## 1. Introduction

Essential oils are complex mixtures of volatile natural compounds, biosynthesized by many aromatic plants. Due to their diverse biological properties, plant volatiles are widely used as food additives to improve flavor and/or taste, and as preservatives to prevent growth of food-borne bacteria and fungi, thereby extending the shelf life of processed foods [1]. In addition to encountering microorganisms acquired resistance to existing preservatives, nowadays, there is a growing consumer demand for organically produced natural food stuff. In this context, the identification and evaluation of natural products, including essential oils, for the development of new classes of natural antimicrobials for food preservation is an important innovative challenge in food science and technology [2].

The Anacardiaceae family is a rich source of these volatile oils, which are isolated from both the fruits and leaves and exhibits a wide spectrum of biological properties [3,4,5]. This family comprises about 70 genera and 700 species with tropical and subtropical distribution. The fruits and pseudo-fruits of many species constitute an important food resource exemplified by cashew (*A. occidentale* L.), mango (*Magifera indica* L.) and pistachio (*Pistacia vera* L.).

The volatile oils from this family of plants are known for their antibacterial activities. For instance, Tassou and Nychas [6] found that the essential oils from *Pistacia* *lentiscus* L. was more active against Gram-positive than Gram-negative bacteria. Volatile oils from leaves of *Schinus* *terebinthifolius* Raddi exhibited antimicrobial activity against both fungi and bacteria [7]. Erazo *et al.* [8] showed that the essential oils from *Schinus polygamus* (Cav.) Cabr., collected in different countries, have diverse compositions and antibacterial activities.

The essential oil produced by *Schinus* *molle* L. demonstrated a preservative effect against *Salmonella anatum* and *S. enteritidis* inoculated into minced beef meat [9]. At low concentrations, this oil revealed bacteriostatic actions. However, at high concentrations, it showed pronounced bactericidal activity. Therefore, the investigation on the biological activities and composition of Anacardiaceae essential oils represents a rational approach for identifying novel substances with potential economic value.

Although the antimicrobial activities of essential oils and their components have been widely reported, their detailed mechanism(s) of action have only been recently studied [1,10,11]. It is envisaged that the mechanisms of action of the volatile oils in the bacterial cell involves the following steps: (i) degradation of the cell wall [12]; (ii) damage to cytoplasmic membrane and their proteins [13]; (iii) leakage of cell contents [12,14,15]; and (iv) depletion of the proton motive force [13]. Volatile oils can also act as pro-oxidants reacting with proteins, membranes and/or DNA through the production of reactive oxygen intermediates (ROI) [11].

However, to the best of our knowledge, oxidative damage within bacterial membranes, in response to volatile oils exposure, has not been previously reported. Consequently, in line with our studies on chemical composition and biological activities of aromatic and medicinal plants [16,17,18,19], herein we describe the chemical composition of essential oils from *A.* *humile* Engl., *A. occidentale* L., *A.* *fraxinifolium* Schott ex Spreng., *M.* *urundeuva* Allemão, and *S.* *terebinthifolius* Raddi and the resultant antibacterial activity of these oils against Gram-positive and Gram-negative bacteria. We also report on the oxidative effects of these oils on bacterial cells by quantifying the extent of lipid peroxidation.

## 2. Results and Discussion

### 2.1. Essential Oil Chemical Compositions

Volatile oil yields from the leaves of the plants investigated ranged from 0.3% w/w to 0.7% w/w. (Table 1). The highest yields were found for *A. fraxinifolium* and *S. terebinthifolius* (0.7% w/w), and the lowest ones for *A. humile* and *A. occidentale* (0.3% w/w) species. As observed in Table 1, the chemical compositions differed widely amongst the species evaluated.

**Table 1:** Constituents and yields of essential oils (%) from species *A. humile*, *A.* *occidentale*, *A.* *fraxinifolium*, *M. urundeuva* and *S.* *terebinthifolius.*

CONSTITUENT	RI *	*A. humile*	*A. occidentale*	*A. fraxinifolium*	*M. urundeuva*	*S. terebinthifolius*	
						March	July
**Hydrocarbon Monoterpenes**		**29.9**	**4.7**	**67.0**	**91.0**	**30.9**	**46.6**
*α*-Pinene	937	22.0 *±* 0.9	-	0.7 *±* 0.0	-	1.2 *±* 0.1	4.2 *±* 0.1
*β*-Pinene	982	6.6 *±* 1.3	-	-	-	-	0.8 *±* 0.0
Myrcene	993	-	-	1.9 *±* 0.1	4.2 *±* 0.3	15.4 *±* 0.9	0.8 *±* 0.0
*δ*-2-Carene	1002	-	-	3.6 *±* 0.1	-	-	-
*α*-Phellandrene	1004	-	-	-	-	-	18.2 *±* 1.2
*δ*-3-Carene	1011	-	-	-	78.8 *±* 1.7	-	-
*ρ*-Cymene	1026	-	-	-	-	-	3.3 *±* 0.2
Limonene	1031	1.3 *±* 0.1	4.7 *±* 0.3	0.4 *±* 0.0	0.2 *±* 0.0	12.0 *±* 0.6	16.7 *±* 1.1
*β*-Phellandrene	1032	-	-	-	3.0 *±* 0.2	-	-
( *Z*)-*β*-Ocimene	1040	-	-	1.1 *±* 0.1	-	-	-
( *E*)-*β*-Ocimene	1050	-	-	44.1 *±* 0.8	-	2.3 *±* 0.2	2.6 *±* 0.1
*α*-Terpinolene	1089	-	-	15.2 *±* 0.9	4.8 *±* 0.6	-	-
**Hydrocarbon Sesquiterpenes**		**60.9**	**80.7**	**19.0**	**8.1**	**59.4**	**35.4**
*δ*-Elemene	1339	-	-	-	-	2.4 *±* 0.1	2.0 *±* 0.0
*α*-Copaene	1376	2.5 *±* 0.3	10.3 *±* 0.9	0.4 *±* 0.0	-	1.7 *±* 0.1	0.6 *±* 0.0
*β*-Elemene	1391	-	-	-	0.7 *±* 0.1	4.8 *±* 0.3	2.1 *±* 0.1
*α*-Gurjunene	1409	1.0 *±* 0.2	-	-	-	-	-
( *E)*-Caryophyllene	1418	31.0 *±* 1.8	15.4 *±* 1.5	-	1.1 *±* 0.1	14.7 *±* 0.8	2.7 *±* 0.2
Aromadendrene	1439	1.1 *±* 0.4	1.1 *±* 0.2	5.6 *±* 0.2	-	-	-
*α*-Humulene	1454	2.9 *±* 0.3	1.5 *±* 0.1	-	-	2.5 *±* 0.1	0.9 *±* 0.0
Alloaromadendrene	1461	1.4 *±* 0.2	2.5 *±* 0.7	0.5 *±* 0.0	-	-	-
*β*-Camigrene	1475	-	1.0 *±* 0.2	-	0.5 *±* 0.0	7.5 *±* 1.0	-
Germacrene-D	1480	5.9 *±* 1.7	11.5 *±* 1.2	1.4 *±* 0.1	-	8.8 *±* 0.3	21.0 *±* 1.2
*β*-Selinene	1485	0.7 *±* 0.0	1.9 *±* 0.3	-	2.5 *±* 0.2	4.3 *±* 0.1	-
*α*-Amorphene	1485	-	3.0 *±* 0.2	0.5 *±* 0.1	-	-	-
Viridiflorene	1493	-	-	9.0 *±* 0.4	3.0 *±* 0.2	-	-
Bicyclogermacrene	1494	7.6 *±* 1.2	8.2 *±* 0.5	-	-	-	-
*δ*-Selinene	1495	-	1.7 *±* 0.2	-	-	-	-
*α*-Muurolene	1499	1.2 *±* 0.4	2.3 *±* 0.1	-	-	2.6 *±* 0.1	0.9 *±* 0.0
Germacrene-A	1503	0.6 *±* 0.1	-	-	0.3 *±* 0.0	2.1 *±* 0.0	1.3 *±* 0.0
( *E*,*E*)-α-Farnesene	1508	-	-	0.8 *±* 0.1	-	-	-
*γ*-Cadinene	1513	0.9 *±* 0.2	2.0 *±* 0.1	0.8 *±* 0.0	-	2.3 *±* 0.1	0.7 *±* 0.0
*δ*-Cadinene	1524	4.1 *±* 0.2	9.3 *±* 0.7	-	-	3.6 *±* 0.1	1.6 *±* 0.1
Germacrene B	1556	-	7.3 *±* 1.1	-	-	2.1 *±* 0.1	1.6 *±* 0.0
**Oxygenated Sesquiterpenes**		**6.3**	**3.8**	**11.6**	**-**	**5.8**	**15.5**
Ledol	1565	-	-	0.5 *±* 0.0	-	-	-
Spathulenol	1576	-	-	5.7 *±* 0.3	-	1.1 *±* 0.0	2.1 *±* 0.1
*β*-Caryophyllene oxide	1581	0.6 *±* 0.0	-	-	-	0.8 *±* 0.0	2.6 *±* 0.1
Globulol	1583	1.4 *±* 0.4	-	3.2 *±* 0.1	-	-	-
Epiglobulol	1588	1.8 *±* 0.2	-	-	-	-	-
Viridiflorol	1590	1.4 *±* 0.3	-	2.2 *±* 0.1	-	-	2.5 *±* 0.2
*δ*-Cadinol	1636	-	1.5 *±* 0.1	-	-	1.3 *±* 0.1	2.4 *±* 0.1
*α*-Muurolol	1645	-	-	-	-	1.2 *±* 0.1	2.8 *±* 0.1
*α*-Cadinol	1653	1.1 *±* 0.2	2.3 *±* 0.2	-	-	1.4 *±* 0.0	3.1 *±* 0.1
**Others**		**-**	**7.2**	**-**	**-**	**-**	**-**
Tetradecane	1399	-	1.7 *±* 0.1	-	-	-	-
Hexadecanoic acid	1984	-	7.2 *±* 1.3	-	-	-	-
**Total**		**97.7**	**96.3**	**97.9**	**99.1**	**96.1**	**97.5**
**Yield (%)**		**0.3** ***±*** **0.0**	**0.3 *±* 0.0**	**0.7 *±* 0.1**	**0.6 *±* 0.0**	**0.6 *±* 0.1**	**0.7 *±* 0.1**

* Retention indexes relative to C_8_–C_27_ linear alkanes series.

The major constituents within *A*. *humile* leaves oil are (*E*)-caryophyllene (31.0%), *α*-pinene (22.0%), bicyclogermacrene (7.6%), *β*-pinene (6.6%) and germacrene-D (5.9%). For the oil from leaves of *A*. *humile* collected in another region from Brazil [20], the authors identified the following major constituents: *α*-bulnesene (8.6%), *γ*-cadinene (7.5%), selina-3,7(11)-diene (6.7%), *α*-himachalene (6.1%) and cyperene (5%). It is not uncommon that different chemical compositions are detected for volatile oils extracted from plant species collected in different locations [21,22]. This difference is also observed upon testing *A. occidentale* oil during the current study. Consequently, the major compounds identified from the leaves collected in Minas Gerais state (Brazil) were (*E*)-caryophyllene (15.4%), germacrene-D (11.5%), *α*-copaene (10.3%), *α*-cadinene (9.3%), bicyclogermacrene (8.2%) and germacrene-B (7.3%). For plants cultivated in Pará state (Brazil), the major compounds are (*E*)-*β*-ocimene (28.8%), *α*-copaene (13.6%), (*E*)-caryophyllene (7.6%) and *δ*-cadinene (9.1%) [23]. It is instructive to compare this result with the oils isolated from plants grown in Nigeria, which were composed mainly of *β*-phellandrene (42.7%), (*E*)-caryophyllene (4.4%), *α*-pinene (4.3%), germacrene-D (4.0%), *p*-cymene (3.2%) and (*E*)-*β*-ocimene (3.1%) [24]. Differences in chemical compositions of these oils are probably associated within genetic variability amongst the populations grown at each location.

The volatile oils produced by *A. fraxinifolium* leaves presented (*E*)-*β*-ocimene (44.1%), *α*-terpinolene (15.2%) and viridiflorene (9.0%) as major constituents. The sesquiterpene hydrocarbons represent about 20% and the oxygenated sesquiterpenes about 12%. In a previous investigation [23], Maia and coworkers found that (*Z*)-*β*-ocimene (42.2%) was the major constituent in leaves from *A*. *fraxinifolium* grown in Mato Grosso state (Brazil), with lower concentrations of bicyclogermacrene (13.3%), limonene (13.2%) and (*E*)-*β*-ocimene (11.1%).

The oils extracted from the leaves of *M. urundeuva* revealed the monoterpene *δ*-3-carene at 78.8% concentration. This monoterpene was also the major component in oils from plants collected in two other states in Brazil, Maranhão (78.1%) and Tocantins (56.3%) [25].

The chemical composition of *S. terebinthifolius* leaves oil in both sampling periods (March and July) confirmed the seasonal variation previously observed for this species [26]. The oil extracted in March presented a high concentration of myrcene (15.4%) and (*E*)-caryophyllene (14.7%) whilst in July, this components represented only 0.8% and 2.7%, respectively of the total oil. Germacrene-D content dropped from 21.0% in July to 8.8% in March, whereas the monoterpene *α*-phellandrene was undetectable in the oils collected in March, but rose to 18.2% in July. In addition, the oils extracted in July contained 15.5% of oxygenated sesquiterpenes compared with only 5.8% in the oils extracted in March.

The essential oils demonstrate a high level of variability in terms of yield and composition and this has been attributed to the interactions between factors such the geographic origin, edaphic and climate features, genetic variability and phenological phase of the plants [27,28,29].

### 2.2. Antibacterial Activity of the Essential Oils

Antibacterial activities of the essential oils were assessed by the agar disc diffusion and the microdilution methods against *S. aureus*, *B. cereus* and *E. coli* (Table 2 and Table 3). The oils of all tested species, except the one from *A. occidentale*, exhibited varying levels of antibacterial activity against Gram-positive and Gram-negative bacteria. In general, the inhibition zones were higher for Gram-positive bacteria (Table 2). Tassou and Nychas [6] also observed a greater effect on Gram-positive organisms for the oil from *P.* *lentiscus* (Anacardiaceae). Similar results were observed by Shimizu *et al.* [30] for the oils from fruits of *Lithraea* *molleoides*.

A relationship between the inhibition zones diameters (Table 2) and the MIC values (Table 3) could not be found. Due to the inherent characteristics of the method, the diffusion coefficients of the different constituents found in essential oils can influence the results. The capacity for each oil constituent to migrate within the agar can markedly influence the size of the inhibition zone. Although this method was standardized by CLSI, it was nevertheless developed for the analysis of conventional antimicrobial agents such as antibiotics. Such drugs are mostly hydrophilic in nature and consequently diffuse more easily in agar, as opposed to essential oils that are volatile, insoluble in water, viscous and possess a complex chemical composition. In addition, for the microdilution method, the oils were dissolved in the broth with the surfactant Tween 80, which may modulate their biological availability and, consequently, their antibacterial activity.

**Table 2:** Diameter of the inhibition zones of bacterial growth for the essential oils from leaves of the species *A. humile*, *A.* *occidentale*, *A.* *fraxinifolium*, *M.* *urundeuva* and *S.* *terebinthifolius.*

Essential oils	Inhibition Zones Diameter (mm) *		
	Gram-negative	Gram-positive	
	*E. coli*	*B. cereus*	*S. aureus*
*A.* *humile*	7 B c	14 A c	10 B d
*A. occidentale*	8 A c	7 A d	8 A d
*A. fraxinifolium*	11 B b	23 A b	13 B c
*M. urundeuva*	14 B b	22 A b	22 A b
*S. terebinthifolius* (March)	6 B c	15 A c	14 A c
*S. terebinthifolius* (July)	14 B b	25 A b	20 A b
Control (H_2_O)	6 A c	6 A d	6 A d
Chloramphenicol 30 µg	29 B a	29 B a	32 A a

* Means followed by same capital letter; lines or minor letter columns are not different by Scott-Knott’s test at *p* 5%.

**Table 3:** Minimum inhibitory concentration (MIC) of bacterial growth for the essential oils extracted from leaves of the species *A.* *fraxinifolium*, *M.* *urundeuva*, *Schinus* *terebinthifolius* and the monoterpene *δ*-3-carene.

Essential Oils/Constituent	Minimum inhibitory Concentration (g L^−1^)		
	Gram-negative	Gram-positive	
	*E. coli*	*B. cereus*	*S. aureus*
*S. terebinthifolius* (March)	0.63	2.50	1.25
*S. terebinthifolius* (July)	0.16	1.25	0.31
*A. fraxinifolium*	0.31	1.25	0.31
*M. urundeuva*	0.31	0.63	0.31
*δ*-3-Carene	0.16	0.16	0.16

Extracts of *S. terebinthifolius* oils prepared in July were more active against all bacterial strains than the oils extracted from plants collected in March (Table 2 and Table 3). The difference in antimicrobial activity is most probably due to the differing chemical composition (Table 1). Therefore, the seasonal variation influences the pharmacological properties of *S. terebinthifolius* oil and this should be carefully regulated when considering its application as an antimicrobial agent.

The region where the plants are grown may also influence the chemical composition of the oils produced. For instance, Erazo *et al.* [8] identified the major component of *S. polygamus* oil collected in Chile as *β*-pinene and this oil proved active against both Gram-positive and Gram-negative bacteria. However, for the oils extracted from plants collected in Argentina, the major constituents were limonene and *α*-phellandrene and this oil was active against Gram-positive *B. cereus*.

Oils extracted from *S. terebinthifolius* leaves collected in July showed elevated oxygenated sesquiterpene concentrations (15.5%) compared with the oils extracted in the month of March (5.8%). The relationship between the oxygenated compounds in the constituents with the higher antimicrobial activity of essential oils has been previously reported. For example, Ultee *et al.* [13] demonstrated that the presence of the phenolic hydroxyl group in carvacrol is essential for its activity against *B. cereus*. Therefore, the higher concentration of oxygenated components observed within oils extracted in July may be related to their greater antibacterial activity. Consequently, *A. fraxinifolium* oil, which also presented a higher concentration of oxygenated compounds, demonstrated high antibacterial activity.

However, oxygenated compounds were absent from *M. urundeuva* oil, but its antibacterial activity was comparable to that of the other oils investigated. The oil of this species was characterized by a high concentration of *δ*-3-carene (78.8%). Some studies do report the effect of this constituent on the tissues and cells of the respiratory system such as broncho-constriction and mucosal irritation [31,32,33], and toxicity to alveolar macrophages [34]. However, to our knowledge, studies on the antibacterial action of *δ*-3-carene remain unreported.

**Figure 1:**
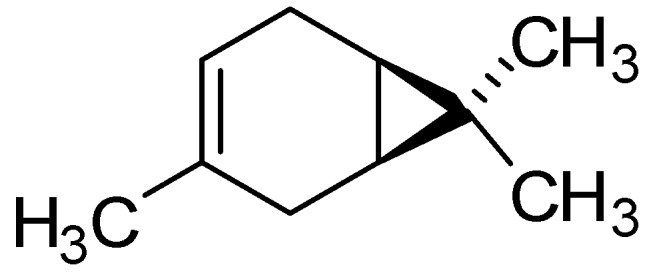
Structural formula of *δ*-3-carene, the major constituent (78.8%) of *M.* *urundeuva* essential oil.

From the data presented in Table 3, it is apparent that *δ-*3-carene is more active itself than the total volatile oils extract. This compound is a hydrocarbon monoterpene (Figure 1) and its hydrophobicity (calculated log*p* = 2.9) enables it to readily partition within the lipids of the bacterial cell membrane, thereby disrupting the structures and rendering them more permeable [1,13]. In conclusion, this compound appears to be a promising lead molecule for analogue synthesis, optimization of which may produce useful new compounds with antibiotic activity.

### 2.3. Lipid Peroxidation within Bacterial Cells

The results presented in Table 4 indicate that essential oils promote an increase in the malondialdehyde (MDA) content within bacterial cells, signaling the process of lipid peroxidation. The highest levels of peroxidation were observed for *S. terebinthifolius* (July) and *M. urundeuva* oils (MDA-TBA_2_ = 2.14 to 3.34 nmol mg^−1^ protein) whereas the lowest effect was seen for the monoterpene *δ*-3-carene.

**Table 4:** MDA-TBA_2_ amounts in bacteria grown in broth supplemented with the MIC concentration for each essential oil extracted from leaves of the species *A.* *fraxinifolium*. *M.* *urundeuva*, *S. terebinthifolius* and the monoterpene *δ*-3-carene.

Essential Oils/Constituents	MDA-TBA_2_ Concentration (nmol mg^−1^ Prot)		
	Gram-negative	Gram-positive	
	*E. coli*	*B.* *cereus*	*S. aureus*
*S. terebinthifolius* (March)	2.16 A b	1.85 B b	1.67 B b
*S. terebinthifolius* (July)	3.34 A a	2.14 B b	2.22 B b
*A. fraxinifolium*	2.46 A b	1.51 B b	1.84 B b
*M. urundeuva*	2.74 A b	2.97 A a	3.21 A a
*δ*-3-Carene	1.33 B c	1.92 A b	1.71 A b
Control	0.69 A d	0.95 A c	0.73 A c

* Means followed by same capital letter; lines or minor letter columns are not different by Scott-Knott’s test at *p* 5%.

In biological membranes, lipid peroxidation is normally associated with oxidative stress and free radical attack and, therefore, generates a complex variety of products, many of which are reactive electrophiles. For instance, malondialdehyde (MDA), a bifunctional tautomer of (*E*)-3-hydroxy-acrylaldehyde, remains the most widely studied among such products [35]. It is formed by successive *β*-scission cascades of peroxidized polyunsaturated fatty acids and is commonly measured by reaction with thiobarbituric acid (TBA) to produce a colored chromogen. This highly reactive *bis*-aldehyde is toxic and interacts with DNA and proteins and it has potentially mutagenic properties [36].

Peroxidation damage of membrane lipids caused by synthetic antimicrobial agents has been described in the literature [37,38]. However, to the best of our knowledge, the effect of volatile oils on lipid peroxidation within bacterial membranes has not been previously reported. Considering the large number of different groups of chemical compounds present in essential oils, it is most likely that their antibacterial activity is not attributable to one specific mechanism and that there are several critical targets in the cell [1,11,39]. Among such targets, the cell membrane is particularly affected because the essential oil components can enter between the fatty acyl chains leading to membrane disruption [40]. This effect was observed to *Bacillus subtilis* treated with *Fortunella crassifolia* essential oil, the cell walls and membranes were partially disintegrated, causing the outflow of cytoplasm [41]. Consequently, our results indicate that the peroxidation of lipid is an important event contributing to the mechanism of antibacterial action of essential oils and deserves more widespread attention.

## 3. Experimental

### 3.1. Plant Material

Aerial parts of *A. humile* Engl., *A. occidentale* L., *A. fraxinifolium* Schott ex Spreng., *M. urundeuva* Allemão, and *Schinus terebinthifolius* Raddi were collected in Minas Gerais state, Brazil. The materials were identified, herborized and voucher specimens were deposited in the VIC Herbarium of the Plant Biology Department, Federal University of Viçosa (Registration Numbers: 31,600, 36,629, 15,709, 20,815 and 30,839). Samples were collected in March 2008. The species *S. terebinthifolius* were collected in the summer (March) and in the winter (July).

### 3.2. Essential Oil Extraction

Leaves were collected in triplicate and in a completely randomized way amongst specimens of the populations investigated. Each sample (100 g) was freshly chopped and subjected to three h hydrodistillation in a Clevenger-type apparatus. The resulting oils were separated from the aqueous phase, weighed and the reported yields were calculated with respect to dry matter mass. Oils were stored under a nitrogen atmosphere and maintained at −4 °C, until they were analyzed by gas chromatography and mass spectrometry and before use within the bioassays reported herein.

### 3.3. Chemical Analysis of the Essential Oil Extraction—GC-FID and GC-MS

GC analyses were carried out with a GC-17A Series instrument (Shimadzu, Japan) equipped with a flame ionization detector (FID). Chromatographic conditions were as follows: fused silica capillary column (30 m × 0.22 mm i.d.) with a DB-5 bonded phase (0.25 μm film thickness); carrier gas, N_2_ at a flow rate of 1.8 mL min^−1^; injector temperature 220 °C, detector temperature 240 °C; column temperature was programmed to start at 55 °C (isothermal for 2 min), with an increase of 3 °C min^−1^ up to 240 °C, isothermal at 240 °C for 15 min; injection of 1.0 μL (1% w/v in dichloromethane); split ratio 1:10; column pressure of 115 kPa. The analyses were carried out in triplicate and the abundance of each compound was expressed as a relative percentage of the total area of the chromatograms.

The GC-MS unit (model GCMS-QP5050A, from Shimadzu) was equipped with a DB-5 fused silica column (30 m × 0.22 mm i.d., film thickness 0.25 µm) and interfaced with an ion trap detector. Transfer line temperature, 240 °C; ion trap temperature, 220 °C; carrier gas, He at a flow rate of 1.8 mL min^−1^; injector temperature 220 °C, detector temperature 240 °C; column temperature was programmed to start at 55 °C (isothermal for 2 min), with an increase of 3 °C min^−1^ up to 240 °C, isothermal at 240 °C for 15 min; injection of 1.0 µL (1% w/v in dichloromethane); split ratio 1:10; column pressure of 100 kPa; ionization energy, 70 eV; scan range, 29–450 u; scan time, 1 s. The identity of each component was assigned by comparison of their retention indexes (RI), relative to a linear alkane standards series (C_8_–C_27_) and also by comparison of its mass spectrum with either reference data from the equipment database (Wiley 7) and from literature sources [42].

### 3.4. Antibacterial Assays

Bacterial strains were obtained from the collections of the Department of Microbiology, Federal University of Viçosa, Viçosa, Minas Gerais state Brazil. Microorganisms used were Gram-positive *Staphylococcus aureus* (ATCC 25923) and *Bacillus cereus*, Ribotype 1 222–173-S4 isolated from post-pasteurization equipment surfaces [43]; and Gram-negative *E. coli* (ATCC 11229).

The agar disc diffusion method was employed to determine the antimicrobial activity of the essential oils, as previously described [44]. Briefly, a suspension of the tested microorganism (2 × 10^8^ CFU mL^−1^), previously activated twice at intervals of 24 h, was spread on Petri plates with Mueller Hinton agar. Filter paper discs (6 mm diameter) were individually impregnated with 5 µL of the essential oils and placed on the inoculated plates. The plates were incubated for 48 h at 37 °C in the cases of *S. aureus* and *E. coli* and at 32 °C for *B. cereus*. The diameters of the inhibition zones were measured using a paquimeter and expressed in millimeters. The antibiotic Chloramphenicol (30 μg) and sterile water were included in this experiment as positive and negative controls. Each test was performed in triplicate and repeated three times. The results were analyzed by ANOVA and Scott-Knott’s multiple-range tests at *p* ≤ 0.05 by using the software GENES (Genetics and Statistical Analysis [45].

A broth microdilution method was used to determine the minimum inhibitory concentration (MIC) for the oil under test [46]. Overnight broth cultures of each strain were prepared in Brain Heart Infusion Broth (Himedia) and the final concentration in each well was adjusted to 2 × 10^5^ CFU/mL following inoculation. The concentration of each inoculum was confirmed by viable count on Plate Count Agar (Himedia). The broth was supplemented with Tween 80 (Merck, Germany) at a concentration of 0.1% (v/v) in order to enhance essential oils solubility. Preliminary control experiments showed that Tween 80 alone did not affect the growth the microorganisms tested. A serial doubling dilution of each essential oil was prepared in a 96-well microtiter plate over the range 0.039 to 5.0 g L^−1^. Positive growth control was prepared in the broth supplemented with 0.1% (v/v) Tween 80. The plates were incubated aerobically for 24 h at 37 °C for *S. aureus*, *E. coli* and lastly at 32 °C for *B. cereus*. The bacterial growth was monitored by turbidity by assaying in a UV spectrophotometer (625 nm) and the MIC was defined as the lowest concentration that reduced the bacterial growth.

### 3.5. Determinations of Lipid Peroxidation Levels

Tubes with 10 mL of Brain Heart Infusion Broth (Himedia), supplemented with 0.1% (v/v) Tween 80 (Merck, Germany), were inoculated with 2 × 10^5^ CFU/mL of bacterial strains. These conditions were used for the control group. Within treatments, the broth was also supplemented with the MIC for each essential oil. After 24 h, the bacterial cells were collected by centrifugation at 10,000 *g* for 10 min. They were washed with 2 mL of 50 mM potassium phosphate buffer at pH 7.5 before being re-suspended in 2.0 mL of this buffer, and the lipid peroxidation was detected by assaying for malondialdehyde (MDA), which reacts with thiobarbituric acid (TBA) [37]. The MDA levels were expressed relative to the concentration of bacterial proteins. Proteins content was determined by the Bradford assay (1976) [47].

## 4. Conclusions

In conclusion, the essential oils from *A.* *fraxinifolium*, *M.* *urundeuva* and *Schinus* *terebinthifolius* leaves, collected in Minas Gerais, Brazil, have promising activity as an organic alternative to commonly used disinfectants and preservatives against both Gram-positive and Gram-negative food-borne bacteria. The volatile oil from *M. urundeuva* is also a potential novel source of the monoterpene *δ*-3-carene. Finally, pro-oxidant damages to the cell membrane appear to be associated with some of the essential oils described herein and may therefore play an important role and so far unrecognized role in the mechanism of antibacterial action of these natural compounds.
